# Synaptic Plasticity and NO-cGMP-PKG Signaling Regulate Pre- and Postsynaptic Alterations at Rat Lateral Amygdala Synapses Following Fear Conditioning

**DOI:** 10.1371/journal.pone.0011236

**Published:** 2010-06-21

**Authors:** Kristie T. Ota, Melissa S. Monsey, Melissa S. Wu, Glenn E. Schafe

**Affiliations:** 1 Department of Psychology, Yale University, New Haven, Connecticut, United States of America; 2 Interdepartmental Neuroscience Program, Yale University, New Haven, Connecticut, United States of America; Medical College of Georgia, United States of America

## Abstract

In vertebrate models of synaptic plasticity, signaling via the putative “retrograde messenger” nitric oxide (NO) has been hypothesized to serve as a critical link between functional and structural alterations at pre- and postsynaptic sites. In the present study, we show that auditory Pavlovian fear conditioning is associated with significant and long-lasting increases in the expression of the postsynaptically-localized protein GluR1 and the presynaptically-localized proteins synaptophysin and synapsin in the lateral amygdala (LA) within 24 hrs following training. Further, we show that rats given intra-LA infusion of either the NR2B-selective antagonist Ifenprodil, the NOS inhibitor 7-Ni, or the PKG inhibitor Rp-8-Br-PET-cGMPS exhibit significant decreases in training-induced expression of GluR1, synaptophysin, and synapsin immunoreactivity in the LA, while those rats infused with the PKG activator 8-Br-cGMP exhibit a significant increase in these proteins in the LA. In contrast, rats given intra-LA infusion of the NO scavenger c-PTIO exhibit a significant decrease in synapsin and synaptophysin expression in the LA, but no significant impairment in the expression of GluR1. Finally, we show that intra-LA infusions of the ROCK inhibitor Y-27632 or the CaMKII inhibitor KN-93 impair training-induced expression of GluR1, synapsin, and synaptophysin in the LA. These findings suggest that the NO-cGMP-PKG, Rho/ROCK, and CaMKII signaling pathways regulate fear memory consolidation, in part, by promoting both pre- and post-synaptic alterations at LA synapses. They further suggest that synaptic plasticity in the LA during auditory fear conditioning promotes alterations at presynaptic sites via NO-driven “retrograde signaling”.

## Introduction

Evidence from both invertebrate and vertebrate model systems has suggested that long-term synaptic plasticity requires N-methyl-D-aspartate receptor (NMDAR)-driven recruitment of intracellular signaling pathways that promote long-term plastic change and memory through alterations of transcription and translation and accompanying morphological changes at both pre- and postsynaptic sites [1], [2], [3], [4], [5], [6]. In *Aplysia*, for example, long-term synaptic facilitation of the gill-withdrawal reflex is thought to be initiated by NMDAR-driven alterations at postsynaptic sites at sensory-motor synapses [7], [8], [9], [10], while signaling via cAMP at presynaptic sites is thought to promote both structural changes [11], [12] and long-term changes in cell excitability [10]. Similarly, long-term potentiation (LTP) in area CA1 of the hippocampus, which is known to be induced via NMDAR-mediated elevations in Ca^2+^ in the postsynaptic cell [1], [13], has been shown to be accompanied not only by postsynaptic morphological alterations [2], [3], but also by corresponding presynaptic changes, including increases in presynaptic vesicle mobilization and release [14], [15] and structural changes in the presynaptic terminal [16], [17].

Studies have suggested that the NO-cGMP-PKG signaling pathway plays a critical role in coordinating these pre- and postsynaptic alterations underlying long-term synaptic plasticity and memory formation [18], [19], [20], [21], [22], [23]. In *in vitro* models of hippocampal synaptic plasticity, NMDAR-driven activation of nitric oxide synthase (NOS) and the formation of nitric oxide (NO) has been suggested to play a critical role in transcriptional regulation and structural plasticity in the postsynaptic cell [22], [24], while, presynaptically, activation of cGMP and protein kinase G (PKG) signaling via “retrograde signaling” of NO has been suggested to promote mobilization of synaptic vesicles and enhanced transmitter release from the presynaptic cell [25] as well as structural changes in the presynaptic terminal [24], [26]. For example, glutamate-induced LTP in hippocampal cell cultures has been shown to promote an increase in the expression of the postsynaptically-localized protein GluR1 and the presynaptically-localized proteins synapsin I and synaptophysin, as well as a corresponding increase in co-localization of GluR1 and synaptophysin/synapsin I-labeled puncta [24]. This increase in LTP-induced clusters of pre- and postsynaptically-localized proteins is impaired by bath application of NMDAR antagonists [24] and inhibitors of NO signaling [26]. In contrast, bath application of exogenous NO or cGMP analogs alone leads to an increase in pre- and postsynaptically-localized protein clusters [26]. Collectively, these findings suggest that NO-cGMP-PKG signaling may be critical for promoting both pre- and postsynaptic aspects of structural plasticity.

While the involvement of NO-cGMP-PKG signaling in structural plasticity has been extensively studied in hippocampal-dependent synaptic plasticity, comparatively few studies have examined whether similar processes underlie amygdala-dependent synaptic plasticity and memory formation. We have recently shown that memory consolidation of auditory Pavlovian fear conditioning and associated synaptic plasticity at thalamic inputs to the lateral amygdala (LA) require NO-cGMP-PKG signaling in the LA [27], [28]. Further, we and others have shown that auditory fear conditioning is associated with pre- and postsynaptic alterations at LA synapses [29], [30], [31], [32]. In the present study, we show that these training-related pre- and postsynaptic changes in the LA are long-lasting and regulated by NMDAR-driven synaptic plasticity and NO-cGMP-PKG signaling at LA synapses.

## Results

### Auditory fear conditioning persistently regulates the expression of the postsynaptically- localized protein GluR1 and the presynaptically-localized proteins synapsin and synaptophysin at LA synapses

Previous *in vitro* work in the hippocampus has suggested that long-term synaptic plasticity is accompanied by both pre- and postsynaptic alterations [2], [3], [4], [5]. Here, we have examined whether auditory fear conditioning promotes pre- and postsynaptic alterations at LA synapses, and whether these effects are long-lasting.

**Figure 1:**
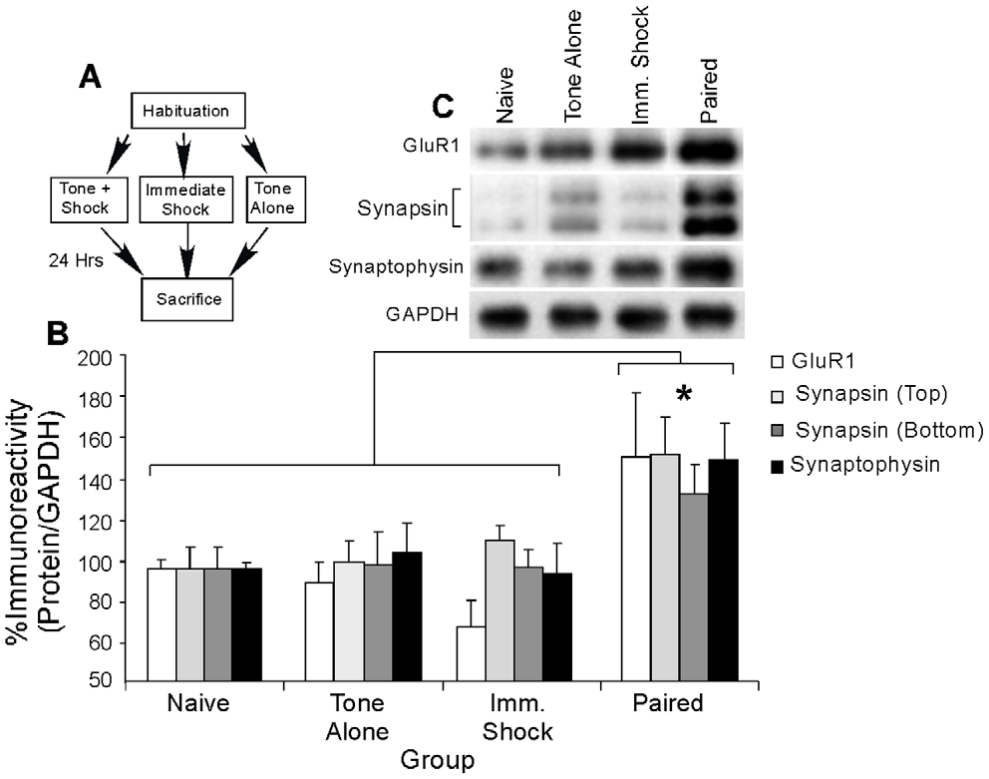
Auditory fear conditioning regulates the expression of pre- and postsynaptically-localized proteins in the LA. (*A*) Schematic of the behavioral protocol. Rats were given either no stimulation (“Naïve”), three tone-alone presentations (“Tone Alone”), three immediate shocks (“Immediate Shock”), or three tone-shock pairings (“Paired”), and sacrificed 24 hours later. (*B*) Mean (±SEM) GluR1, Synapsin (bottom and top bands), and Synaptophysin immunoreactivity from LA punches taken from Naïve (*n* = 6), Tone Alone (*n* = 8), Immediate Shock (*n* = 8), or Paired (*n* = 7) animals. Here, GluR1, Synapsin (bottom and top bands), and Synaptophysin protein levels have been normalized to GAPDH levels for each sample and expressed as a percentage of the Naïve group. (*C*) Representative blots for GluR1, Synapsin (bottom and top bands), Synaptophysin, and GAPDH in the LA. (*) *p*<0.05 relative to Paired rats.

In our first series of experiments, we examined whether auditory fear conditioning regulates the expression of the postsynaptically-localized protein GluR1 [33] and the presynaptically-localized Ca^2+^-regulated synaptic vesicle proteins synapsin and synaptophysin [34] at LA synapses (Figure 1). Rats were exposed to either no stimulation (“Naive”), tone alone (“Tone Alone”), immediate shock (“Imm. Shock”), or paired presentations of tone and shock (“Paired”), followed by sacrifice 24 hours after conditioning (Figure 1a). We then used Western blotting on punches taken from the LA to examine whether auditory fear conditioning regulates the expression of GluR1, synapsin, and synaptophysin in the LA.

Figure 1b depicts immunolabeling for each of the synaptic proteins in Paired, Tone Alone, and Imm. Shock groups relative to Naïve controls, while representative Western blots can be viewed in Figure 1c. We analyzed the data using an overall Group by Protein ANOVA and found a significant effect of Group [F(3,101) = 15.72, p<0.0001]; the effect for Protein [F(3,101) = 0.43] and the Group by Protein interaction [F(9,101) = 0.29] were not significant. Post hoc analysis using Duncan's t-tests showed that the Paired group exhibited significantly higher expression of GluR1, synapsin, and synaptophysin relative to the Naïve (p<0.001), Tone Alone (p<0.001), or Immediate Shock groups (p<0.001). Furthermore, levels of the loading control, GAPDH, did not differ between the Naïve, Tone Alone, Immediate Shock, and Paired groups (p>0.05; not shown). These results suggest that auditory fear conditioning, but not presentation of tone or shock alone, regulates pre- and postsynaptic changes at LA synapses.

**Figure 2:**
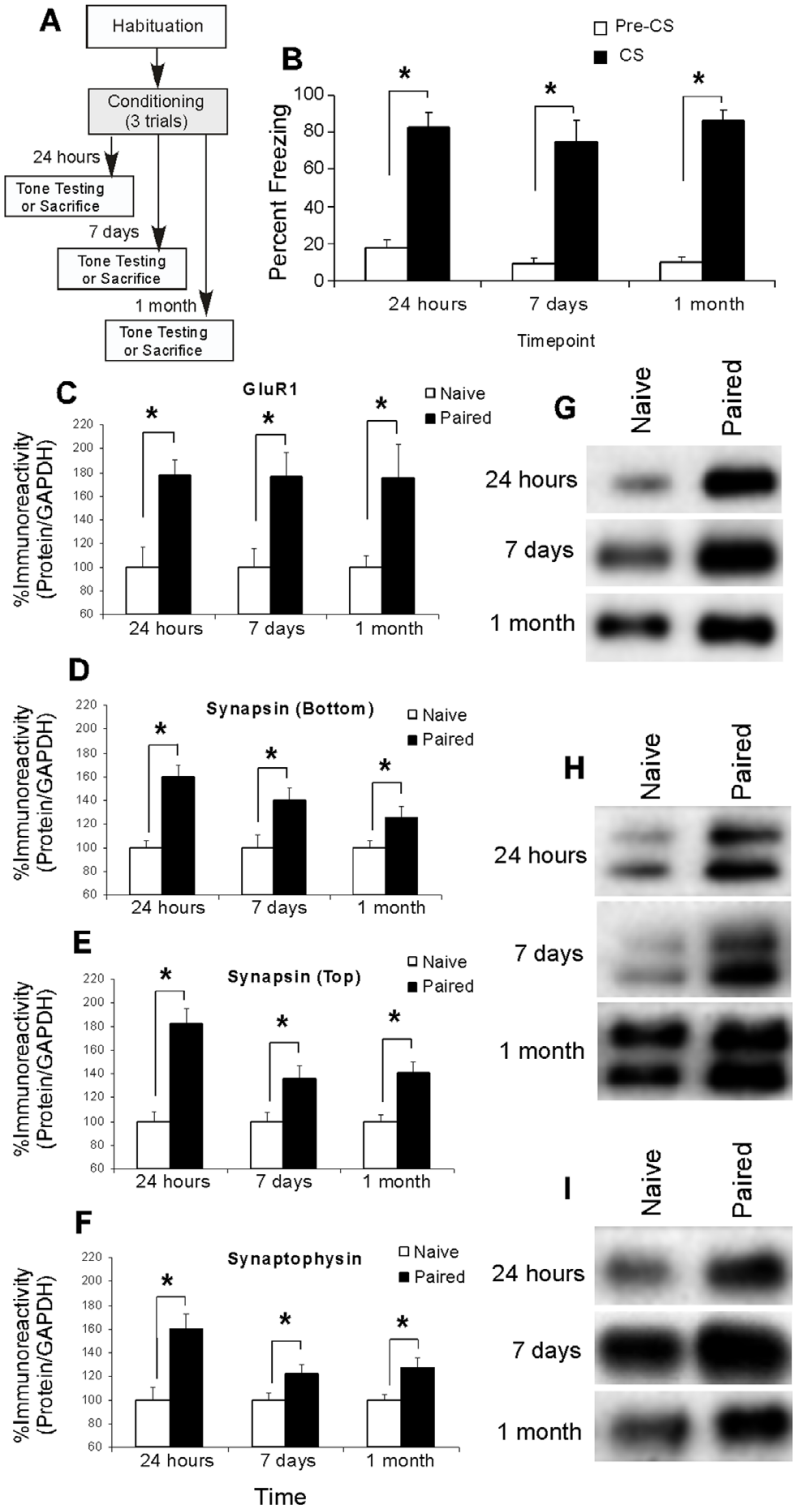
Auditory fear conditioning results in long-lasting auditory fear memory retention and persistent regulation of the expression of pre- and postsynaptically-localized proteins in the LA. (*A*) Schematic of the behavioral protocol. Separate groups of rats were trained with three tone-shock pairings, then either tested for retention of auditory fear conditioning or sacrificed at 24 hours, 7 days, or 1 month following conditioning. (*B*) Mean (± SEM) freezing scores for pre-CS and CS periods assessed at 24 hours (*n* = 6), 7 days (*n* = 6), or 1 month (*n* = 5) following conditioning. (*) *p*<0.05 relative to pre-CS freezing levels. (*C*) Mean (±SEM) GluR1 immunoreactivity from LA punches taken from Naïve (24 hours, *n* = 8; 7 days, *n* = 7; 1 month, *n* = 8) or Paired (24 hours, *n* = 9; 7 days, *n* = 7; 1 month, *n* = 8) groups. (*) *p*<0.05 relative to Naïve rats. (*D*) Mean (±SEM) Synapsin (bottom band) immunoreactivity from LA punches taken from Naïve (24 hours, *n* = 8; 7 days, *n* = 7; 1 month, *n* = 8) or Paired (24 hours, *n* = 9; 7 days, *n* = 7; 1 month, *n* = 8) groups. (*E*) Mean (±SEM) Synapsin (top band) immunoreactivity from LA punches taken from Naïve (24 hours, *n* = 8; 7 days, *n* = 7; 1 month, *n* = 8) or Paired (24 hours, *n* = 9; 7 days, *n* = 7; 1 month, *n* = 8) groups. (*) *p*<0.05 relative to Naïve rats. (*F*) Mean (±SEM) percent Synaptophysin immunoreactivity from LA punches taken from Naïve (24 hours, *n* = 8; 7 days, *n* = 7; 1 month, *n* = 8) or Paired (24 hours, *n* = 9; 7 days, *n* = 7; 1 month, *n* = 8) groups. (*) *p*<0.05 relative to Naïve rats. In each experiment, protein levels for GluR1, synapsin, or synaptophysin were normalized to GAPDH levels for each sample and expressed as a percentage of the Naïve group. (*G*) Representative blots for GluR1 in the LA at 24 hours, 7 days, or 1 month following conditioning. (*H*) Representative blots for Synapsin (bottom and top bands) in the LA at 24 hours, 7 days, or 1 month following conditioning. (*I*) Representative blots for Synaptophysin in the LA at 24 hours, 7 days, or 1 month following conditioning.

We next examined whether these training-induced alterations in pre- and postsynaptically-localized proteins at LA synapses are long-lasting. Separate groups of rats were exposed to either no stimulation (“Naïve”) or tone-shock pairings (“Paired”), and then sacrificed at either 24 hours, 7 days, or 1 month following conditioning (Figure 2a). Western blotting on tissue taken from the LA was performed to assay training-induced expression of GluR1, synapsin, and synaptophysin in the LA at each of the different time points. In separate groups of rats, we examined memory retention at 24 hrs, 7 days, or 1 month following auditory fear conditioning (Figure 2a).

Rats tested for auditory fear memory 24 hours following conditioning were found to have intact fear memory retention, as revealed by comparison of freezing during the pre-CS period relative to that of the CS period [t(5) = 10.76; p<0.001] (Fig. 2b). Fear memory to the tone was also intact at 7 days [t(5) = 6.37; p<0.01] and 24 days after training [t(4) = 10.97; p<0.001] (Fig. 2b). These results indicate that our conditioning protocol promotes auditory fear memory formation that persists for up to 1 month following training.

The findings of our Western blot analyses are presented in Figure 2 c–f. Relative to naïve controls, fear conditioned rats exhibited significant increases in levels of GluR1 expression in the LA at 24 hours [t(15) = 3.87, p<0.01], 7 days [t(12) = 2.66, p<0.05], and 1 month [t(14) = 2.45, p<0.05] following fear conditioning (Figure 2c). Similarly, fear conditioned rats exhibited significant long-lasting increases in levels of synapsin (Figure 2 d–e) and synaptophysin (Figure 2f) in the LA. Analysis of synapsin revealed a significant increase at 24 hrs [bottom band: t(15) = 5.50, p<0.001; top band: t(15) = 5.35, p<0.001], 7 days [bottom band: t(12) = 2.48, p<0.05; top band: t(12) = 2.55, p<0.05], and 1 month [bottom band: t(14) = 2.25, p<0.05; top band: t(14) = 3.77, p<0.01] following fear conditioning. Analysis of synaptophysin revealed a significant increase at 24 hrs [t(15) = 3.77, p<0.01], 7 days [t(12) = 2.24, p<0.05], and 1 month [t(14) = 2.63, p<0.05] following fear conditioning. Representative Western blots for GluR1, synapsin, and synaptophysin can be viewed in Figures 2 g–i, respectively.

Importantly, levels of the loading control, GAPDH, did not differ between naïve and trained rats at 24 hours, 7 days, or 1 month following conditioning for any of these samples (p>0.05; not shown), indicating that overall protein levels were not significantly changed in the trained animals relative to naïve controls. Taken together, this series of experiments suggests that auditory fear conditioning regulates the expression of pre- and postsynaptically-localized proteins in the LA, and that these changes are long-lasting.

### Synaptic plasticity and NO-cGMP-PKG signaling regulate training-induced alterations in pre- and postsynaptically-localized proteins at LA synapses following fear conditioning

**Figure 3:**
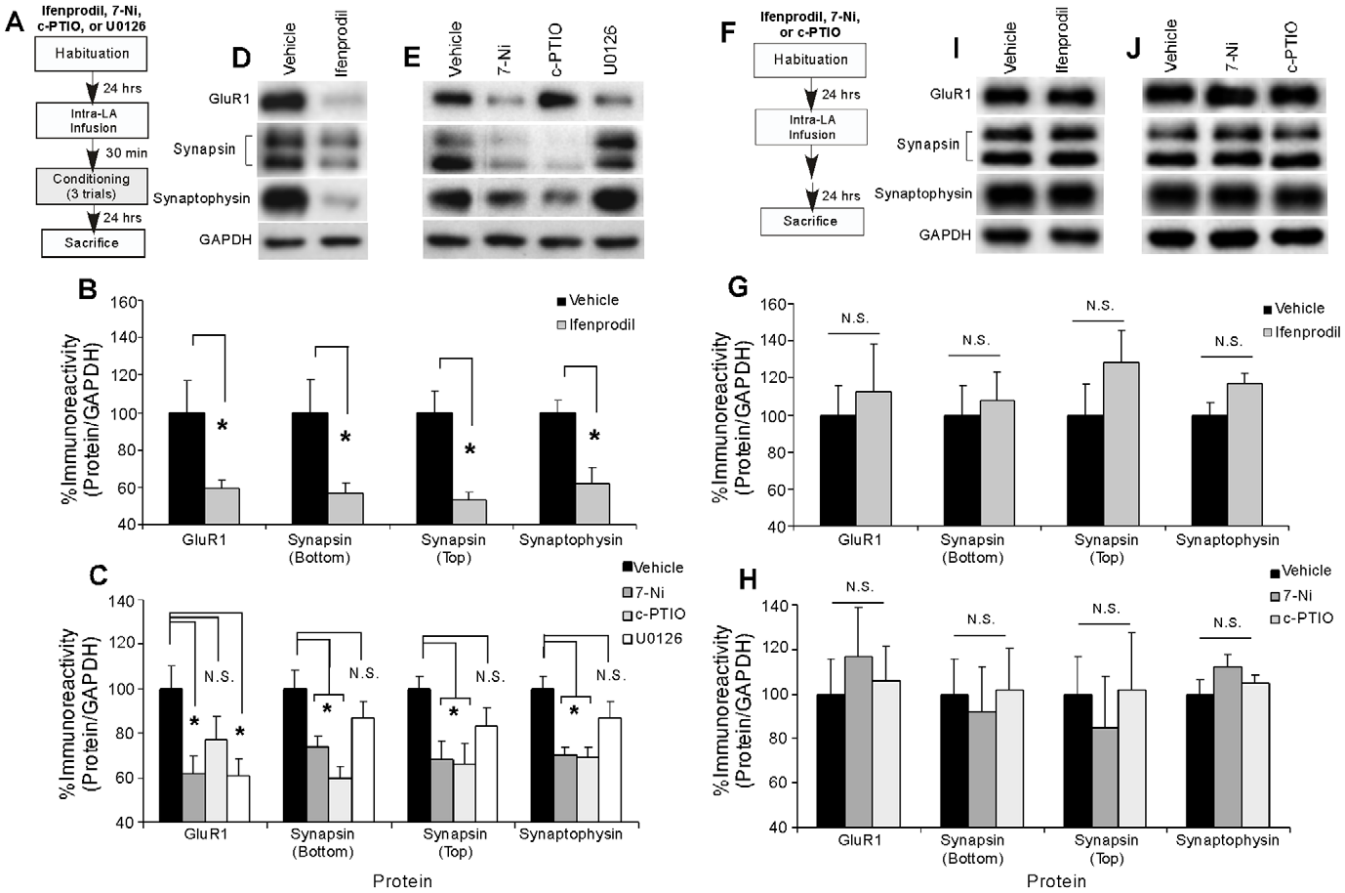
Synaptic plasticity and the NO signaling pathway regulate training-induced alterations in pre- and postsynaptically-localized proteins in the LA following fear conditioning. (*A*) Schematic of the behavioral protocol. Rats were given intra-LA infusion of vehicle, Ifenprodil (1µg/side), 7-Ni (1µg/side), c-PTIO (1µg/side), or U0126 (1µg/side), trained, and then sacrificed 24 hours later. (*B*) Mean (±SEM) GluR1, Synapsin (bottom and top bands), and Synaptophysin immunoreactivity from LA punches taken from rats given intra-LA infusion of vehicle (*n* = 8) or Ifenprodil (*n* = 8). (*) *p*<0.05 relative to vehicle-infused rats. (*C*) Mean (±SEM) GluR1, Synapsin (bottom and top bands), and Synaptophysin immunoreactivity from LA punches taken from rats given intra-LA infusion of vehicle (*n* = 8), 7-Ni (*n* = 9), c-PTIO (*n* = 6), or U0126 (*n* = 9). (*) *p*<0.05 relative to vehicle-infused rats. In each experiment, protein levels for GluR1, synapsin, or synaptophysin were normalized to GAPDH levels for each sample and expressed as a percentage of the Vehicle-infused group. (*D*) Representative blots for GluR1, Synapsin (bottom and top bands), Synaptophysin, and GAPDH in the LA for the Ifenprodil experiment. (*E*) Representative blots for GluR1, Synapsin (bottom and top bands), Synaptophysin, and GAPDH in the LA for the 7-Ni, c-PTIO, and U0126 experiment. (*F*) Schematic of the behavioral protocol. Naïve rats were given intra-LA infusion of vehicle, Ifenprodil (1 µg/side), 7-Ni (1 µg/side), or c-PTIO (1 µg/side) followed by sacrifice 24 hours later. (*G*) Mean (±SEM) GluR1, Synapsin (bottom and top bands), and Synaptophysin immunoreactivity from LA punches taken from naïve rats given intra-LA infusions of vehicle (GluR1: *n* = 4; Synapsin bottom band: *n* = 6, Synapsin top band: *n* = 6, Synaptophysin: *n* = 6) or Ifenprodil (GluR1: *n* = 5; Synapsin bottom band: *n* = 6, Synapsin top band: *n* = 6, Synaptophysin: *n* = 6). (*H*) Mean (±SEM) GluR1, Synapsin (bottom and top bands), and Synaptophysin immunoreactivity from LA punches taken from naïve rats given intra-LA infusions of vehicle (GluR1: *n* = 4; Synapsin bottom band: *n* = 6, Synapsin top band: *n* = 6, Synaptophysin: *n* = 6), 7-Ni (GluR1: *n* = 5; Synapsin bottom band: *n* = 6, Synapsin top band: *n* = 6, Synaptophysin: *n* = 6), or c-PTIO (GluR1: *n* = 5; Synapsin bottom band: *n* = 6, Synapsin top band: *n* = 6, Synaptophysin: *n* = 6). In each experiment, protein levels for GluR1, synapsin, or synaptophysin were normalized to GAPDH levels for each sample and expressed as a percentage of the Vehicle-infused group. (*I*) Representative blots for GluR1, Synapsin (bottom and top bands), Synaptophysin, and GAPDH in the LA for the Ifenprodil naïve experiment. (*J*) Representative blots for GluR1, Synapsin (bottom and top bands), Synaptophysin, and GAPDH in the LA for the 7-Ni and c-PTIO naïve experiment.

**Figure 4:**
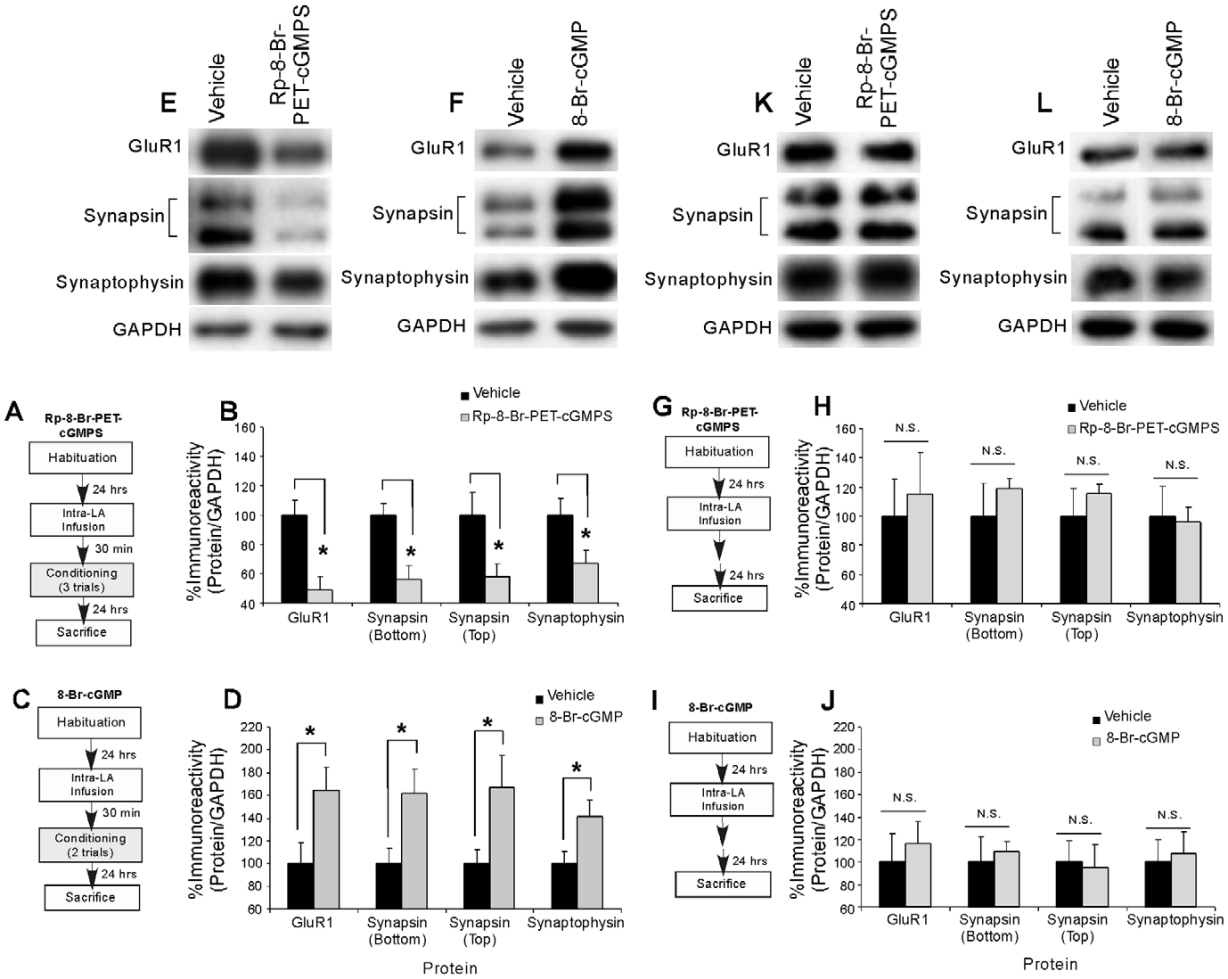
The NO-cGMP-PKG signaling pathway regulates training-induced alterations in pre- and postsynaptically-localized proteins in the LA following fear conditioning. (*A*) Schematic of the behavioral protocol. Rats were given intra-LA infusion of vehicle or Rp-8-Br-PET-cGMPS (1 µg/side), trained, and then sacrificed 24 hours later. (*B*) Mean (±SEM) GluR1, Synapsin (bottom and top bands), and Synaptophysin immunoreactivity from LA punches taken from rats given intra-LA infusions of vehicle (*n* = 8) or Rp-8-Br-PET-cGMPS (*n* = 8). (*) *p*<0.05 relative to vehicle-infused rats. (*C*) Schematic of the behavioral protocol. Rats were given intra-LA infusion of vehicle or 8-Br-cGMP (10 µg/side), trained, and then sacrificed 24 hours later. (*D*) Mean (±SEM) GluR1, Synapsin (top and bottom bands), and Synaptophysin immunoreactivity from LA punches taken from rats given intra-LA infusion of vehicle (*n* = 8) or 8-Br-cGMP (*n* = 8). (*) *p*<0.05 relative to vehicle-infused rats. In each experiment, protein levels for GluR1, synapsin, or synaptophysin were normalized to GAPDH levels for each sample and expressed as a percentage of the Vehicle-infused group. (*E*) Representative blots for GluR1, Synapsin (bottom and top bands), Synaptophysin, and GAPDH in the LA for the Rp-8-Br-PET-cGMPS experiment. (*F*) Representative blots for GluR1, Synapsin (bottom and top bands), Synaptophysin, and GAPDH in the LA for the 8-Br-cGMP experiment. (*G*) Schematic of the behavioral protocol. Naive rats were given intra-LA infusion of vehicle or Rp-8-Br-PET-cGMPS (1 µg/side) followed by sacrifice 24 hours later. (*H*) Mean (±SEM) GluR1, Synapsin (bottom and top bands), and Synaptophysin immunoreactivity from LA punches taken from naïve rats given intra-LA infusion of vehicle (GluR1: *n* = 4; Synapsin bottom band: *n* = 5, Synapsin top band: *n* = 5, Synaptophysin: *n* = 5) or Rp-8-Br-PET-cGMPS (GluR1: *n* = 4; Synapsin bottom band: *n* = 5, Synapsin top band: *n* = 5, Synaptophysin: *n* = 5). (*I*) Schematic of the behavioral protocol. Rats were given intra-LA infusion of the vehicle or 8-Br-cGMP (10 µg/side), then sacrificed 24 hours later. (*J*) Mean (±SEM) GluR1, Synapsin (bottom and top bands), and Synaptophysin immunoreactivity from LA punches taken from naïve rats given intra-LA infusion of vehicle (GluR1: *n* = 4; Synapsin bottom band: *n* = 5, Synapsin top band: *n* = 5, Synaptophysin: *n* = 5) or 8-Br-cGMP (GluR1: *n* = 4; Synapsin bottom band: *n* = 5, Synapsin top band: *n* = 5, Synaptophysin: *n* = 5). In each experiment, protein levels for GluR1, synapsin, or synaptophysin were normalized to GAPDH levels for each sample and expressed as a percentage of the Vehicle-infused group. (*K*) Representative blots for GluR1, Synapsin (bottom and top bands), Synaptophysin, and GAPDH in the LA for the Rp-8-Br-PET-cGMPS naïve experiment. (*L*) Representative blots for GluR1, Synapsin (bottom and top bands), Synaptophysin, and GAPDH in the LA for the 8-Br-cGMP naïve experiment.

In our first set of experiments, we showed that pre- and postsynaptically-localized proteins at LA synapses are persistently regulated following auditory Pavlovian fear conditioning. Here, we examined whether NMDAR-driven synaptic plasticity and NO-cGMP-PKG signaling in the LA regulate these pre- and postsynaptic alterations at LA synapses following fear learning. In the first series of experiments, rats were given intra-LA infusion of either vehicle, the NR2B selective antagonist Ifenprodil (1 µg/side; 0.5 µL), the NOS inhibitor 7-Ni (1 µg/side; 0.5 µL), the membrane impermeable NO scavenger c-PTIO (1 µg/side; 0.5 µL), or the MEK inhibitor U0126 (1 µg/side; 0.5 µL). In the second series of experiments, rats were given intra-LA infusion of the PKG inhibitor Rp-8-Br-PET-cGMPS (1 µg/side; 0.5 µL) or the PKG activator 8-Br-cGMP (10 µg/side; 0.5 µL). The dose of Ifenprodil has previously been shown to significantly impair fear memory acquisition when infused into the LA prior to fear conditioning [35]. The doses of c-PTIO, 7-Ni, U0126, and Rp-8-Br-PET-cGMPS have been shown to significantly impair fear memory consolidation when infused into the LA prior to fear conditioning; that is, fear acquisition and short-term memory (STM) are intact, while long-term memory (LTM) is impaired [27], [28], [36]. Conversely, the dose of 8-Br-cGMP has recently been shown to significantly enhance fear memory consolidation when infused into the LA prior to fear conditioning [28]. Following infusion, rats were trained, followed by sacrifice 24 hours later (see Methods for details; Figures 3a, 4a,c). Western blotting on tissue taken from around the cannula tips in the LA was then performed to determine whether training-induced activation of GluR1, synapsin, and synaptophysin at LA synapses is regulated by intra-LA infusion of each of these drugs.

The findings of the Ifenprodil experiment are depicted in Figure 3b, while representative Western blots can be viewed in Figure 3d. Relative to vehicle-infused controls, rats given intra-LA infusions of Ifenprodil prior to training exhibited significant decreases in levels of GluR1 [t(14) = 2.28, p<0.05], synapsin [bottom band: t(14) = 2.34, p<0.05; top band: t(14) = 3.88, p<0.01], and synaptophysin [t(14) = 3.67, p<0.01] protein expression in the LA. In addition, levels of the loading control, GAPDH, did not differ between the vehicle and Ifenprodil-infused groups for any of the proteins (p>0.05; not shown). Importantly, this reduction in pre- and postsynaptic protein expression in the LA following intra-LA infusion of Ifenprodil was not observed in naïve animals that did not receive fear conditioning (Figure 3g,i), indicating that the effects of Ifenprodil on pre- and postsynaptic protein expression in the LA are not due to a general, non-specific effect of infusion of the drug alone. Relative to vehicle controls, naive rats given intra-LA infusion of Ifenprodil prior to sacrifice at the same time as the trained animals described above exhibited no significant differences in levels of GluR1, synapsin, or synaptophysin in the LA [GluR1: t(7) = 0.40, p>0.05; synapsin (bottom band): t(10) = 0.34, p>0.05; synapsin (top band): t(10) = 1.14, p>0.05; synaptophysin: t(10) = 2.02, p>0.05]. In addition, levels of the loading control, GAPDH, did not differ between the naïve groups for any of the proteins (p>0.05; not shown). Taken together, these findings suggest that NMDAR-driven synaptic plasticity in the LA regulates alterations in pre- and postsynaptically-localized protein expression at LA synapses following fear conditioning.

The findings of the 7-Ni, c-PTIO, and U0126 experiment are depicted in Figure 3c, while representative Western blots may be viewed in Figure 3e. Rats given intra-LA infusion of either vehicle, 7-Ni, c-PTIO, or U0126 prior to training exhibited significant decreases in levels of GluR1 [F(3,28) = 4.06, p<0.05], synapsin [bottom band: F(3,28) = 5.86, p<0.01; top band: F(3,28) = 5.02, p<0.05], and synaptophysin [F(3,28) = 6.06, p<0.01] protein expression in the LA. Post hoc t-tests further revealed interesting differences between drug groups. Analysis of GluR1 revealed that the vehicle-infused group differed significantly from those infused with either 7-Ni (p<0.05) or U0126 (p<0.05), but not from that infused with c-PTIO (p>0.05). Conversely, analysis of synapsin revealed that the vehicle-infused group differed significantly from groups infused with either 7-Ni (bottom band: p<0.05; top band: p<0.05) or c-PTIO (bottom band: p<0.01; top band: p<0.05), but not from that infused with U0126 (bottom band: p>0.05; top band: p>0.05). A similar picture emerged following analysis of synaptophysin, with vehicle-infused animals differing significantly from those infused with either 7-Ni (p<0.01) or c-PTIO (p<0.01), but not from those infused with U0126 (p>0.05). Furthermore, levels of the loading control, GAPDH, did not differ between any of the groups (p>0.05; not shown). Importantly, these alterations in pre- and postsynaptic protein expression in the LA following intra-LA infusion of 7-Ni or c-PTIO were not observed in naïve animals that did not receive fear conditioning (Figure 3h,j). Relative to vehicle controls, naive rats given intra-LA infusion of 7-Ni or c-PTIO prior to sacrifice at the same time as the trained animals described above exhibited no significant differences in levels of GluR1, synapsin, or synaptophysin in the LA [GluR1: F(2,11) = 0.22, p>0.05; synapsin (bottom band): F(2,15) = 0.07, p>0.05; synapsin (top band): F(2,15) = 0.18, p>0.05; synaptophysin: F(2,15) = 1.27, p>0.05]. In addition, levels of the loading control, GAPDH, did not differ between any of the naïve groups for any of the proteins (p>0.05; not shown).

The findings of the Rp-8-Br-PET-cGMPS experiment are depicted in Figure 4b, while the findings of the 8-Br-cGMP experiment are depicted in Figure 4d. Representative Western blots from each of these experiments can be viewed in Figure 4 e–f, respectively. Relative to vehicle-infused controls, rats given intra-LA infusion of Rp-8-Br-PET-cGMPS prior to training exhibited significant decreases in levels of GluR1 [t(14) = 3.87, p<0.01], synapsin [bottom band: t(14) = 3.65, p<0.01; top band: t(14) = 2.38, p<0.05], and synaptophysin [t(14) = 2.36, p<0.05] protein expression in the LA. In addition, levels of the loading control, GAPDH, did not differ between the vehicle and Rp-8-Br-PET-cGMPS-infused groups (p>0.05; not shown). This reduction in pre- and postsynaptic protein expression in the LA following intra-LA infusion of Rp-8-Br-PET-cGMPS was not observed in naïve animals that did not receive fear conditioning (Figure 4h,k). Relative to vehicle controls, naive rats given intra-LA infusion of Rp-8-Br-PET-cGMPS prior to sacrifice at the same time as the trained animals described above exhibited no significant differences in levels of GluR1, synapsin, or synaptophysin in the LA [GluR1: t(6) = 0.37, p>0.05; synapsin (bottom band): t(8) = 0.82, p>0.05; synapsin (top band): t(8) = 0.82, p>0.05; synaptophysin: t(8) = 0.17, p>0.05]. In addition, levels of the loading control, GAPDH, did not differ between naïve groups for any of the proteins (p>0.05; not shown).

In contrast to the Rp-8-Br-PET-cGMPS findings, rats given intra-LA infusion of the PKG activator 8-Br-cGMP prior to training exhibited significant increases in levels of GluR1 [t(14) = 2.36, p<0.05], synapsin [bottom band: t(14) = 2.49, p<0.05; top band: t(14) = 2.20, p<0.05], and synaptophysin [t(14) = 2.26, p<0.05] protein expression in the LA (Figure 4d). In addition, levels of the loading control, GAPDH, did not differ between the vehicle and 8-Br-cGMP-infused groups (p>0.05; not shown). Further, this enhancement in pre- and postsynaptic protein expression in the LA following intra-LA infusion of 8-Br-cGMP was not observed in naïve animals that did not receive fear conditioning (Figure 4j,l). Relative to vehicle controls, naive rats given intra-LA infusion of 8-Br-cGMP prior to sacrifice at the same time as the trained animals described above exhibited no significant differences in levels of GluR1, synapsin, or synaptophysin in the LA [GluR1: t(6) = 0.48, p>0.05; synapsin (bottom band): t(8) = 0.36, p>0.05; synapsin (top band): t(8) = 0.17, p>0.05; synaptophysin: t(8) = 0.22, p>0.05]. In addition, levels of the loading control, GAPDH, did not differ between naïve groups for any of the proteins (p>0.05; not shown).

Collectively, these findings suggest that NMDAR-driven synaptic plasticity, NO-cGMP-PKG signaling, and ERK/MAPK activation regulate the expression of pre- and postsynaptically-localized proteins at LA synapses following fear conditioning, but in unique ways. Blockade of NMDAR-driven synaptic plasticity via Ifenprodil or of NOS activation via 7-Ni impairs training-induced expression of both pre-and postsynaptically-localized proteins in the LA. In contrast, blockade of extracellular NO release via c-PTIO selectively impairs training-induced regulation of presynaptically-localized proteins, while having no significant effect on the postsynaptically- localized protein GluR1. Conversely, blockade of ERK activation via U0126 selectively impairs training-induced regulation of GluR1, while having no effect on that of the two presynaptically- localized proteins.

### The Rho/ROCK and CaMKII signaling pathways regulate training-induced alterations in pre- and postsynaptically-localized proteins at LA synapses following fear conditioning

**Figure 5:**
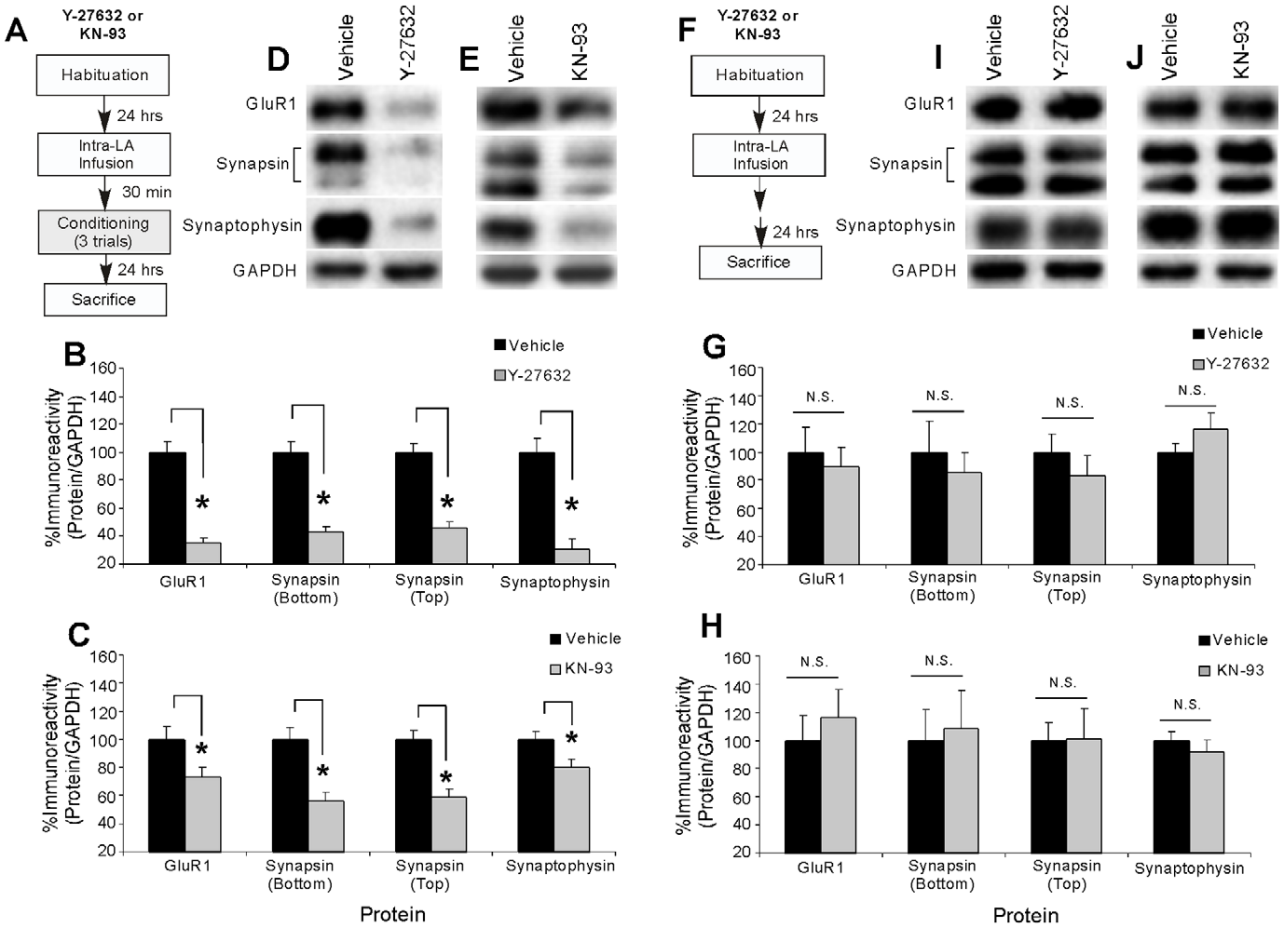
The Rho/ROCK and CaMKII pathways regulate training-induced alterations in pre- and postsynaptically-localized proteins in the LA following fear conditioning. (*A*) Schematic of the behavioral protocol. Rats were given intra-LA infusion of the vehicle, Y-27632 (8.56 µg/side), or KN-93 (1 µg/side), trained, and then sacrificed 24 hours later. (*B*) Mean (±SEM) GluR1, Synapsin (bottom and top bands), and Synaptophysin immunoreactivity from LA punches taken from rats given intra-LA infusions of vehicle (*n* = 8) or Y-27632 (*n* = 9). (*) *p*<0.05 relative to vehicle-infused rats. (*C*) Mean (±SEM) GluR1, Synapsin (bottom and top bands), and Synaptophysin immunoreactivity from LA punches taken from rats given intra-LA infusion of vehicle (GluR1, *n* = 8; Synapsin bottom band, *n* = 7; Synapsin top band, *n* = 7; Synaptophysin, *n* = 8) or KN-93 (GluR1, *n* = 9; Synapsin bottom band, *n* = 7; Synapsin top band, *n* = 7; Synaptophysin, *n* = 9). (*) *p*<0.05 relative to vehicle-infused rats. In each experiment, protein levels for GluR1, synapsin, or synaptophysin were normalized to GAPDH levels for each sample and expressed as a percentage of the Vehicle-infused group. (*D*) Representative blots for GluR1, Synapsin (bottom and top bands), Synaptophysin, and GAPDH in the LA for the Y-27632 experiment. (*E*) Representative blots for GluR1, Synapsin (bottom and top bands), Synaptophysin, and GAPDH in the LA for the KN-93 experiment. (*F*) Schematic of the behavioral protocol. Naive rats were given intra-LA infusion of the vehicle, Y-27632 (8.56 µg/side), or KN-93 (1 µg/side) followed by sacrifice 24 hours later. (*G*) Mean (±SEM) GluR1, Synapsin (bottom and top bands), and Synaptophysin immunoreactivity from LA punches taken from naïve rats given intra-LA infusion of vehicle (*n* = 6) or Y-27632 (*n* = 6). (*H*) Mean (±SEM) percent GluR1, Synapsin (top and bottom bands), and Synaptophysin immunoreactivity from LA punches taken from naïve rats given intra-LA infusion of vehicle (*n* = 6) or KN-93 (*n* = 6). In each experiment, protein levels for GluR1, synapsin, or synaptophysin were normalized to GAPDH levels for each sample and expressed as a percentage of the Vehicle-infused group. (*I*) Representative blots for GluR1, Synapsin (bottom and top bands), Synaptophysin, and GAPDH in the LA for the Y-27632 naïve experiment. (*J*) Representative blots for GluR1, Synapsin (bottom and top bands), Synaptophysin, and GAPDH in the LA for the KN-93 naïve experiment.

Thus far, we have shown that NMDAR-driven synaptic plasticity and NO-cGMP-PKG signaling regulate pre- and postsynaptic changes at LA synapses following auditory fear conditioning. In this final series of experiments, we examined the involvement of the Rho/ROCK and CaMKII signaling pathways in regulating these pre- and postsynaptic modifications at LA synapses. *In vitro* studies in hippocampal area CA1 have shown that LTP-induced alterations in pre- and postsynaptic proteins are blocked by inhibitors of actin polymerization [24] and associated with increases in the phosphorylation of the actin cytoskeleton regulators VASP and RhoA [26]. Further, recent findings suggest that presynaptically localized αCaMKII is a critical substrate for LTP induced by the NO-cGMP-PKG signaling pathway. Presynaptic injection of an αCaMKII inhibitor peptide blocks both LTP and accompanying presynaptic morphological alterations induced by an NO donor or cGMP analog [37], suggesting that αCaMKII may act downstream of cGMP-PKG signaling to promote presynaptic aspects of plasticity in CA1. To examine whether each of these signaling pathways might be involved in the pre- and postsynaptic alterations at LA synapses accompanying fear conditioning, we gave rats intra-LA infusion of the ROCK inhibitor Y-27632 (8.56 µg/side; 0.5 µL) or the CaMKII inhibitor KN-93 (1 µg/side; 0.5 µL), doses which have been shown to significantly impair fear memory formation [38] (see also Figure S1 and Supplemental Methods & Analysis S1). Following infusion, rats were trained, then sacrificed 24 hours after training as in our previous experiments, and Western blotting was performed on LA homogenates to determine whether training-induced activation of GluR1, synapsin, and synaptophysin at LA synapses is regulated by intra-LA infusion of each of these drugs (Figure 5a).

The findings of the ROCK inhibitor experiment can be viewed in Figure 5b, while representative Western blots can be seen in Figure 5d. Relative to vehicle-infused controls, rats given intra-LA infusions of Y-27632 prior to training exhibited significant decreases in levels of GluR1 [t(15) = 7.48, p<0.001], synapsin [bottom band: t(15) = 6.54, p<0.001; top band: t(15) = 6.99, p<0.001], and synaptophysin [t(15) = 5.77, p<0.001] protein expression in the LA. In addition, levels of the loading control, GAPDH, did not differ between the vehicle and Y-27632-infused groups (p>0.05; not shown). This reduction in pre- and postsynaptic protein expression in the LA following intra-LA infusion of Y-27632 was not observed in naïve animals that did not receive fear conditioning (Figure 5g,i). Relative to vehicle controls, naive rats given intra-LA infusion of Y-27632 prior to sacrifice at the same time as the trained animals described above exhibited no significant differences in levels of GluR1, synapsin, or synaptophysin in the LA [GluR1: t(10) = 0.46, p>0.05; synapsin (bottom band): t(10) = 0.55, p>0.05; synapsin (top band): t(10) = 0.85, p>0.05; synaptophysin: t(10) = 1.21, p>0.05]. In addition, levels of the loading control, GAPDH, did not differ between the naïve groups for any of the proteins (p>0.05; not shown). Taken together, these results suggest that changes in the actin cytoskeleton conferred by the Rho/ROCK signaling pathway may be responsible, at least in part, for the pre- and postsynaptic changes detected at the LA synapse following fear conditioning.

The findings of the CaMKII inhibitor experiment can be viewed in Figure 5c, while representative Western blots can be seen in Figure 5e. Relative to vehicle-infused controls, rats given intra-LA infusions of KN-93 prior to training exhibited significant decreases in levels of GluR1 [t(15) = 2.30, p<0.05], synapsin [bottom band: t(12) = 3.83, p<0.01; top band: t(12) = 4.62, p<0.001], and synaptophysin [t(15) = 2.51, p<0.05] protein expression in the LA. In addition, levels of the loading control, GAPDH, did not differ between the vehicle and KN-93-infused groups (p>0.05; not shown). Further, this reduction in pre- and postsynaptic protein expression in the LA following intra-LA infusion of KN-93 was not observed in naïve animals that did not receive fear conditioning (Figure 5h,j). Relative to vehicle controls, naive rats given intra-LA infusion of KN-93 prior to sacrifice at the same time as the trained animals described above exhibited no significant differences in levels of GluR1, synapsin, or synaptophysin in the LA [GluR1: t(10) = 0.59, p>0.05; synapsin (bottom band): t(10) = 0.21, p>0.05; synapsin (top band): t(10) = 0.04, p>0.05; synaptophysin: t(10) = 0.79, p>0.05]. In addition, levels of the loading control, GAPDH, did not differ between the naïve groups for any of the proteins (p>0.05; not shown).

Collectively, these results suggest that both the Rho/ROCK and CaMKII signaling pathways regulate pre- and postsynaptic changes at LA synapses following fear conditioning, possibly via acting as a downstream target of NMDAR-driven synaptic plasticity and the NO-cGMP-PKG signaling pathway.

## Discussion

*In vitro* models of synaptic plasticity have suggested that NMDAR-driven recruitment of intracellular signaling pathways promote long-term plastic change and memory through alterations of transcription and translation and accompanying morphological changes at both pre- and postsynaptic sites [1], [2], [3], [4], [5], [6]. Further, studies have suggested that the NO-cGMP-PKG signaling pathway and “retrograde signaling” via NO play a critical role in coordinating these two events [18], [19], [20], [21], [22], [23]. In the present study, we show that auditory Pavlovian fear conditioning is associated with significant and persistent increases in the expression of the postsynaptically-localized protein GluR1 and the presynaptically-localized proteins synapsin and synaptophysin in the LA. Further, we show that these pre-and postsynaptic alterations at LA synapses are regulated by NMDAR-driven synaptic plasticity and signaling via the NO-cGMP-PKG, Rho/ROCK, and CaMKII pathways in the LA.

Several recent studies have suggested that amygdala-dependent Pavlovian fear conditioning is characterized by both pre- and postsynaptic alterations at LA synapses. Fear conditioning and associated synaptic plasticity in the LA, for example, have been shown to promote the insertion of new AMPA receptors into postsynaptic spines of LA neurons [29], [30]. Further, fear conditioning promotes the transcription of genes involved in cytoskeletal remodeling in LA neurons, including the CRE-mediated gene NF-1 [39], and interference with molecular pathways known to be involved in structural plasticity during early development, such as the Rho-GAP signaling pathway, have been shown to disrupt memory formation in the LA [38]. Fear conditioning has also been shown to drive actin cytoskeleton–regulatory proteins, such as profilin, into amygdala spines shortly after training [40], and to be accompanied by an increase in spinophilin-immunoreactive dendritic spines in the LA [41]. Finally, a recent study has shown that fear conditioning leads to an increase in the expression of the presynaptically- localized protein synaptophysin at LA synapses [31]. Each of these studies, however, has examined alterations in structural plasticity in the LA at relatively short intervals following fear conditioning (e.g. ≤24 hrs); longer time points were not examined. In our study, we found training-induced alterations in pre- and postsynaptically-localized proteins in the LA not only at 24 hours, but also at 7 days and 1 month following fear conditioning. This long-lasting change in pre- and postsynaptically localized proteins in the LA is consistent with both our current data and previous research showing the enduring role of the LA in Pavlovian fear conditioning [42] and suggests that persistent alterations in structural plasticity at LA synapses underlie long-term fear memory formation.

Our findings are consistent with a large body of *in vitro* evidence which suggests that synaptic plasticity in vertebrate models of memory formation involves both pre- and postsynaptic alterations coordinated by extracellular signaling. Of particular relevance to the present manuscript, LTP induced by glutamate application in hippocampal cell cultures has been observed to lead to an increase in GluR1, synaptophysin, and synapsin I labeled puncta, as well as a corresponding increase in the number of sites where GluR1 and synaptophysin/synapsin I are co-localized [24]. This increase in clusters of pre- and postsynaptically-localized proteins is blocked by bath application of the NMDAR antagonists APV or MK-801 [24] and by inhibitors of NO signaling [26]. Conversely, application of exogenous NO or cGMP analogs alone induces LTP and promotes an increase in clusters of both GluR1 and synaptophysin/synapsin I puncta [26]. Remarkably, these LTP-induced alterations in pre- and postsynaptic proteins occur very rapidly in culture (within 5 min) and are independent of protein synthesis [24]. In the present study, we have observed increases in GluR1, synapsin, synaptophysin in the LA at 24 hrs (and at longer time points) following fear conditioning; we did not examine the regulation of these proteins at shorter intervals. However, when considered collectively with the *in vitro* findings it is tempting to speculate that there may be two phases of structural plasticity following fear learning – one leading to rapid, protein synthesis-independent increases in clusters of pre- and postsynaptic proteins, and another leading to more permanent, protein synthesis-dependent modifications at LA synapses.

**Figure 6:**
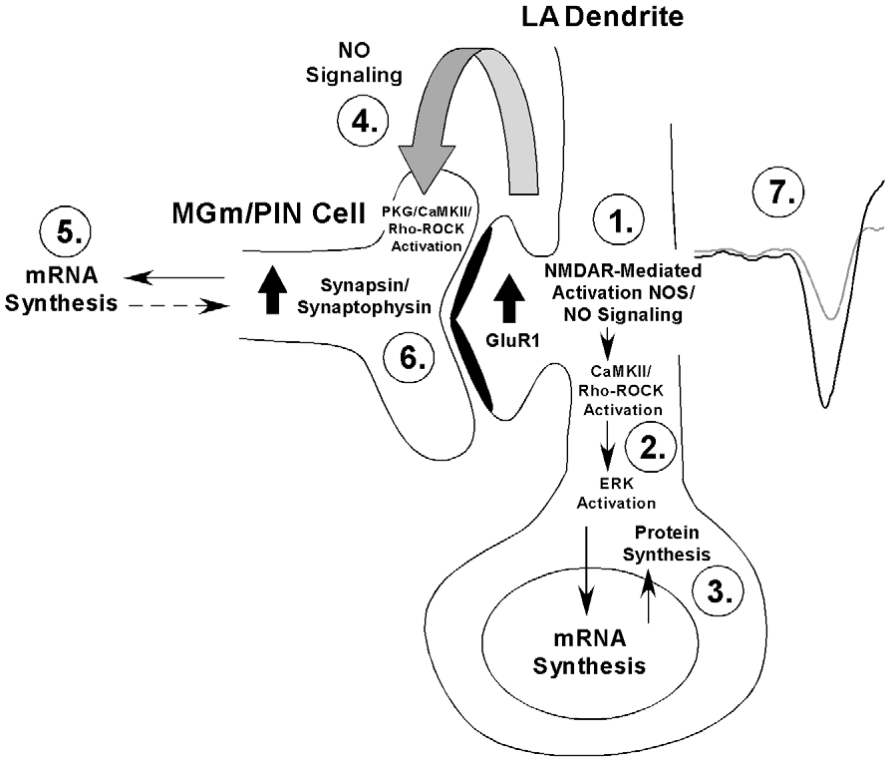
A model of fear memory consolidation. Fear memory consolidation is hypothesized to involve both pre- and postsynaptic modifications at LA synapses (here depicted at the thalamo-LA synapse). These modifications are first triggered by NMDAR-mediated activation of NO-cGMP-PKG and other protein kinase signaling pathways that ultimately promote transcription in LA neurons (Steps 1–3) and resultant postsynaptic functional and/or structural changes that contribute to the formation of the memory. Second, NMDAR-driven activation of NOS in LA neurons is hypothesized to lead to the extracellular release of NO (Step 4), which can in turn promote protein kinase activation and, ultimately, transcriptional changes in MGm/PIN neurons (Step 5) that contribute to presynaptic functional and/or structural changes at LA synapses (Step 6). Together with the postsynaptic modifications in the LA, these presynaptic modifications act to strengthen the connectivity of thalamo-LA synapses, which is reflected neurophysiologically in an enhanced response to the CS in the LA after training (Step 7). See text for details.

We have recently shown that blockade or facilitation of the NO-cGMP-PKG signaling pathway in the LA impairs or enhances memory consolidation of Pavlovian fear conditioning and synaptic plasticity in the LA by activating the ERK/MAPK signaling pathway, suggesting that NO-driven increases in ERK-driven transcriptional regulation in the LA regulate the formation of long-term memory storage [27], [28]. Interestingly, recent work in our lab has pointed to a critical role of ERK-driven transcription in fear memory consolidation not only in the LA, but also in regions of the auditory thalamus that are presynaptic to the LA, including the medial division of the medial geniculate and posterior intralaminar nuclei (MGm/PIN) [32], [43]. Specifically, we have shown that ERK [43] and ERK-driven gene expression [32] in the MGm/PIN are required for fear memory consolidation, and that NMDAR-driven synaptic plasticity and NO signaling in the LA at the time of fear learning coordinately regulate ERK-driven transcriptional changes in both LA and MGm/PIN neurons [32], [44]. In parallel to the findings of the present study, rats given intra-LA infusion of Ifenprodil, 7-Ni, or Rp-8-Br-PET-cGMPS exhibit significant decreases in training-induced expression of phosphorylated ERK and the ERK-driven immediate early genes Arc/Arg3.1, EGR-1, and c-Fos in both the LA and the MGm/PIN, while those rats infused with 8-Br-cGMP exhibit a significant increase in the same proteins at both sites [44]. Further, rats given intra-LA infusion of c-PTIO exhibit a significant decrease in ERK phosphorylation and ERK-driven gene expression in the MGm/PIN, but not in the LA [44]. Furthermore, our findings suggest that the functional significance of LA-driven alterations in ERK signaling and ERK-driven gene expression in the MGm/PIN is to promote presynaptic aspects of plasticity back at the level of the LA. In support of this hypothesis, we have shown that intra-MGm/PIN infusion of a MEK inhibitor blocks synaptic plasticity in the LA [43] and, more recently, that intra-MGm/PIN infusion of an antisense oligodeoxynucleotide to EGR-1 impairs the training-induced expression of synapsin and synaptophysin at LA synapses [32]. When considered collectively with the findings in the present study, this pattern of findings is consistent with a revised model of fear memory consolidation which suggests that synaptic plasticity and the NO-cGMP-PKG signaling pathway regulate fear memory consolidation, in part, by promoting both pre- and postsynaptic changes at thalamo-LA synapses (Figure 6). They further suggest that synaptic plasticity at thalamo-LA synapses during fear conditioning promotes alterations at presynaptic sites via NO-driven “retrograde signaling” [32].

In our final series of experiments, we show that intra-LA infusion of pharmacological inhibitors of the CaMKII and the Rho/ROCK signaling pathways significantly impairs training-induced alterations at pre- and postsynaptic sites at LA synapses, suggesting that each of these targets may act downstream of the NO-cGMP-PKG signaling pathway to promote structural plasticity in the LA. *In vitro* models of synaptic plasticity in the hippocampus have suggested a role for CaMKII as a pre- and postsynaptic target of PKG. Presynaptic injection of a membrane impermeable CaMKII inhibitor peptide, for example, has been shown to block both LTP at hippocampal CA1 synapses and accompanying presynaptic morphological alterations induced by NO donors or a PKG activator [37], suggesting that CaMKII may act downstream of cGMP-PKG signaling to promote presynaptic aspects of plasticity. Further, fear conditioning has been shown to regulate the autophosphorylation of αCaMKII at postsynaptic sites, while inhibition of CaMKII activity in the LA impairs fear memory formation and synaptic plasticity at thalamo-LA synapses [45]. Additional studies will be required to determine whether auditory fear conditioning similarly regulates CaMKII at presynaptic sites in the LA, and whether this is driven by NO-cGMP-PKG signaling.

The Rho/ROCK signaling pathway has also been implicated as a target of NO-cGMP-PKG signaling during synaptic plasticity. Both the vasodilator-stimulated phosphoprotein (VASP) and the small GTPase RhoA have been shown to be downstream substrates of PKG [46], [47] and are thought to regulate the actin cytoskeleton [48], leading to modifications of synaptic plasticity both pre- and postsynaptically [26]. For example, studies employing hippocampal cultures found that LTP-induced alterations in pre- (synapsin and synaptophysin) and postsynaptic (GluR1) proteins were associated with increases in the phosphorylation of the actin cytoskeleton regulators VASP and RhoA [26], and impaired following application of an actin polymerization inhibitor [24]. In addition, an inhibitor of the RhoA-dependent kinase (ROCK), which is activated by RhoGTPase [49], has been shown to impair glutamate-induced LTP in hippocampal cell cultures [26]. These findings suggest that the Rho/ROCK signaling pathway may be critical for both pre- and postsynaptic aspects of structural plasticity. Of particular interest to the present study, VASP was found to have increased levels of phosphorylation at Ser-239 [26], a site that is preferentially phosphorylated by PKG [50], suggesting that VASP phosphorylation may be PKG-dependent. Importantly, the Rho-ROCK signaling pathway has been implicated in memory formation of auditory fear conditioning. For instance, intra-LA infusion of the ROCK inhibitor Y-27632, at the same dose used in the present study, has been shown to impair fear memory consolidation; that is, LTM is impaired, while STM is intact [38]. Collectively, these findings suggest that the Rho/ROCK signaling pathway may promote fear memory formation via alterations in structural plasticity at pre- and postsynaptic sites. Additional studies will be required to determine whether auditory fear conditioning similarly regulates Rho/ROCK signaling at presynaptic sites in the LA, and whether this is driven by NO-cGMP-PKG signaling.

In summary, the findings of the present study suggest that auditory Pavlovian fear conditioning promotes enhanced, long-lasting alterations in pre- and postynaptically localized proteins at LA synapses that are regulated by NMDAR-driven synaptic plasticity, NO-cGMP-PKG signaling, and the Rho/ROCK and CaMKII signaling pathways. These findings define a biochemical mechanism whereby intracellular signaling pathways in the LA at the time of fear learning may induce changes at both sides of LA synapses. These findings make an additional contribution towards understanding the cellular and molecular processes underlying emotional memory formation in the mammalian brain, and provide further support for the hypothesis that NO signaling serves as a “retrograde messenger” in mammalian memory formation.

## Materials and Methods

### Subjects

Adult male Sprague-Dawley rats (Harlan) were housed individually in plastic cages and maintained on a 12∶12 hr light/dark cycle. Food and water were provided *ad libitum* throughout the experiment.

### Drugs

The NR2B selective antagonist Ifenprodil (Sigma, Cat. No. 12892) was dissolved in physiological saline and 2% HBC to yield a stock solution of 2µg/µL. The NOS inhibitor 7-Ni (EMD Chemicals, Cat. No. 483400), the membrane impermeable NO scavenger Carboxy-PTIO (c-PTIO; Tocris, Cat. No. 0772), and the MEK inhibitor U0126 (Promega, Cat. No. V1121) were dissolved in 100% DMSO to yield a stock concentration of 4 µg/µl, which was then diluted 1∶1 in ACSF prior to infusion. The PKG inhibitor Rp-8-Br-PET-cGMPS (Calbiochem, Cat. No. 370679), the CaMKII inhibitor KN-93 (Calbiochem, Cat. No. 422711), and the PKG activator 8-Br-cGMP (Calbiochem, Cat. No. 203820) were dissolved in distilled water for a stock concentration of either 2µg/µL (Rp-8-Br-PET-cGMPS and KN-93) or 20µg/µL (8-Br-cGMP). The ROCK inhibitor Y-27632 (Calbiochem, Cat. No. 688000) was dissolved in ACSF for a stock concentration of 17.12µg/µL.

### Surgical procedures

Under a mixture of Ketamine (100 mg/kg) and Xylazine (6.0 mg/kg) anesthesia, rats were implanted bilaterally with 26- (for Y-27632 and KN-93) or 22-gauge (for all other pharmacological experiments) stainless steel guide cannulas (Plastics One) aimed at the LA. The coordinates for the LA were: −3.2 mm, ±5.0 mm, −8.0 mm relative to Bregma. The guide cannulas were fixed to screws in the skull using a mixture of acrylic and dental cement, and a 31- or 28-gauge dummy cannula was inserted into each guide cannula to prevent clogging. Rats were given Buprenex (0.2 mg/kg) as an analgesic and given at least five days to recover prior to experimental procedures. All procedures were conducted in accordance with the National Institutes of Health *Guide for the Care and Use of Experimental Animals* and were approved by the Yale University Animal Care and Use Committee.

### Fear conditioning experiments

Rats were habituated to handling and to the conditioning boxes (except for the immediate shock group, to prevent learning of the context) for four days prior to training. On the training day, “Paired” rats were given 3 conditioning trials consisting of a 20 sec, 5kHz, 75dB tone that co-terminated with a 1.0 sec, 1mA foot shock (ITI = 120 sec). Controls received either 3 presentations of the tone alone (“Tone Alone”) or 3 immediate shocks upon being introduced to the conditioning chamber (“Imm. Shock”). This immediate shock procedure allows the experimenter to assess the effect of the shock alone on gene expression, in the absence of a context-shock association [51]. “Naïve” rats received no stimulation on the training day and remained in their home cages. Twenty-four hours following training, animals were transferred to the laboratory in their home cages and sacrificed using an overdose of chloral hydrate (600 mg/kg) and decapitated. In other experiments, rats received 3 conditioning trials consisting of a 20 sec, 5kHz, 75dB tone that co-terminated with a 1.0 sec, 1mA foot shock (ITI = 120 sec) and were sacrificed either twenty-four hours, 7 days, or 1 month following training. Brains were frozen and stored at −80°C until processed.

### Pharmacological experiments

Rats were habituated to handling and dummy cannula removal for two days prior to training. On the training day, animals were given an intra-LA infusion of either ACSF [vehicle; containing (in mM): 115 NaCl, 3.3 KCl, 1 MgSO_4_, 2 CaCl_2_, 25.5 NaHCO_3_, 1.2 NaH_2_PO_4_, and 10 glucose], the NR2B selective antagonist Ifenprodil (1 µg/side in 0.5 µL; 0.25 µL/min), the PKG inhibitor Rp-8-Br-PET-cGMPS (1 µg/side in 0.5 µL; 0.25 µL/min), the PKG activator 8-Br-cGMP (10 µg/side in 0.5 µL; 0.25 µL/min), the ROCK inhibitor Y-27632 (8.56µg/side in 0.5 µL; 0.25 µL/min), or the CaMKII inhibitor KN-93 (1 µg/side in 0.5 µL; 0.25 µL/min). In other experiments, rats received intra-LA infusion of 50% DMSO in ACSF (vehicle), the NOS inhibitor 7-Ni (1 µg/side in 0.5 µL; 0.25 µL/min), the membrane impermeable NO scavenger c-PTIO (1 µg/side in 0.5 µL; 0.25 µL/min), or the MEK inhibitor U0126 (1 µg/side in 0.5 µL; 0.25 µL/min). Injectors remained in the cannulas for 1 minute after drug infusion to allow diffusion of the drug from the tip. Thirty (Ifenprodil, 7-Ni, c-PTIO, U0126, Y-27632, KN-93 and respective controls) or sixty (Rp-8-Br-PET-cGMPS, 8-Br-cGMP, and respective controls) minutes following drug infusion, rats were trained with 2 (8-Br-cGMP) or 3 (all other drugs) conditioning trials consisting of a 20 sec, 5kHz, 75dB tone that co-terminated with a 1.0 sec, 0.5 mA or 1 mA foot shock, respectively (ITI = 120 sec). For experiments involving intra-LA infusion of 8-Br-cGMP, rats were trained with 2 tone-shock pairings with a 0.5 mA shock intensity in an effort to avoid ceiling effects that might obscure observation of training-induced elevations in GluR1, synaptophysin, and synapsin protein above the level of vehicle controls [28]. Twenty-four hours following training, animals were sacrificed using an overdose of chloral hydrate (600 mg/kg) and decapitated. Brains were frozen and stored at −80°C until processed.

### Western blotting

For Western blotting, punches containing the LA were obtained using a 1 mm punch tool (Fine Science Tools, Foster City, CA) from 400-µm-thick sections taken on a sliding freezing microtome. Punches were manually dounced in 100µl of ice-cold hypotonic lysate buffer [10 mM Tris-HCl, pH 7.5, 1 mM EDTA, 2.5 mM sodium pyrophosphate, 1mM phenylmethylsulfonyl fluoride, 1 mM β-glycero-phosphate, 1% Igepal CA-630, 1% protease inhibitor cocktail (Sigma) and 1 mM sodium orthovanadate]. Sample buffer (25µl) was immediately added to the homogenates, and the samples were boiled for 4 min. Homogenates were electrophoresed on 10% Tris-HCl gels and blotted to Immobilon-P (Millipore, Bedford, MA). Western blots were blocked in 5% milk in TTBS buffer (50 mM Tris-HCl, pH 7.5, 150 mM NaCl, and 0.05% Tween 20) then incubated with anti-GluR1 (1∶1000; AbCam), anti-Synapsin (1∶1000; Cell Signaling), or anti-Synaptophysin (1∶5000; DakoCytomation) antibody. Blots were then incubated with anti-rabbit conjugated to horseradish peroxidase (1∶20K; Cell Signaling) and developed using enhanced chemiluminescence (Pierce). We analyzed the two isoforms of synapsin (“bottom band” and “top band”) separately. GAPDH (1∶5000; Abcam) was used as a loading control for all Western blotting experiments to control for inconsistencies in protein loading. Optical densities of the bands were analyzed using NIH Image software.

### Behavioral Experiments

For two days prior to conditioning, rats were habituated to handling and to the conditioning boxes. On the training day, they were given 3 conditioning trials consisting of a 20 sec, 5kHz, 75dB tone that co-terminated with a 1.0 sec, 1mA foot shock (ITI = 120 sec). Testing for conditioned fear to the tone occurred at 24 hours, 7 days, or 1 month following training in separate groups of rats. For each test, rats were placed in a distinctive environment that was dark and consisted of a flat black plastic floor that had been washed with a peppermint-scented soap, and they were exposed to 3 conditioned stimulus (CS) tones (5kHz, 75dB, 20 sec). For each tone test, we measured the rats' freezing behavior, defined as a lack of all movement with the exception of that required for respiration, and expressed this measure as a percentage of the total CS presentation time. Freezing was calculated from activity counts measured automatically during each CS presentation by Coulbourne Instruments Activity Monitors (Model # H10-24A) mounted at the top of each of the behavioral chambers. For each memory test, freezing scores during the CS were averaged across trials for each rat, and these were compared to similarly averaged pre-CS freezing scores for each rat. Data was analyzed with repeated-measure t-tests. Differences were considered significant if *p*<0.05.

## Supporting Information

Figure S1 Inhibition of CaMKII in the LA impairs the acquisition, but not expression, of auditory fear memory. (A) Schematic of the behavioral protocol. Rats were given intra-LA infusion of either the vehicle or KN-93 (1 ug). Thirty minutes later they were trained with five tone-shock pairings, then tested for retention of auditory fear conditioning at 3 and 24 hrs following conditioning. Twenty-four hours after the LTM test, rats that had originally been infused with vehicle were re-infused with either ACSF (n = 3) or 1 ug KN-93 (n = 4), then re-tested for auditory fear memory 30 minutes later. (B)Mean (+/− SEM) post-shock freezing between conditioning trials in rats given intra-LA infusions of ACSF (vehicle; n = 7) or 1 ug KN-93 (n = 8). (C) Mean (±SEM) auditory fear memory assessed at 3 hr (STM) and 24 hrs (LTM) following conditioning. (D) Mean (±SEM) auditory fear memory assessed at 30 minutes following re-infusion. Histological verification of cannula placements for rats infused with 1 ug KN-93 (white circles) or ACSF vehicle (black circles). Panels adapted from Paxinos and Watson (1997). (*) p<0.05 relative to vehicle.
(9.24 MB TIF)

Methods & Analysis S1 Supplemental methods and analysis for Figure S1.
(0.03 MB DOC)

## References

[1] Malenka RC, Nicoll RA (1999). Long-term potentiation–a decade of progress?. Science.

[2] Engert F, Bonhoeffer T (1999). Dendritic spine changes associated with hippocampal long-term synaptic plasticity.. Nature.

[3] Toni N, Buchs PA, Nikonenko I, Bron CR, Muller D (1999). LTP promotes formation of multiple spine synapses between a single axon terminal and a dendrite.. Nature.

[4] Bonhoeffer T, Staiger V, Aertsen A (1989). Synaptic plasticity in rat hippocampal slice cultures: local “Hebbian” conjunction of pre- and postsynaptic stimulation leads to distributed synaptic enhancement.. Proc Natl Acad Sci U S A.

[5] Lisman JE, Harris KM (1993). Quantal analysis and synaptic anatomy–integrating two views of hippocampal plasticity.. Trends Neurosci.

[6] Roberts AC, Glanzman DL (2003). Learning in Aplysia: looking at synaptic plasticity from both sides.. Trends Neurosci.

[7] Lin XY, Glanzman DL (1994). Hebbian induction of long-term potentiation of Aplysia sensorimotor synapses: partial requirement for activation of an NMDA-related receptor.. Proc R Soc Lond B Biol Sci.

[8] Murphy GG, Glanzman DL (1996). Enhancement of sensorimotor connections by conditioning-related stimulation in Aplysia depends upon postsynaptic Ca2+.. Proc Natl Acad Sci U S A.

[9] Murphy GG, Glanzman DL (1997). Mediation of classical conditioning in Aplysia californica by long-term potentiation of sensorimotor synapses.. Science.

[10] Antonov I, Antonova I, Kandel ER, Hawkins RD (2003). Activity-dependent presynaptic facilitation and hebbian LTP are both required and interact during classical conditioning in Aplysia.. Neuron.

[11] Bailey CH, Montarolo P, Chen M, Kandel ER, Schacher S (1992). Inhibitors of protein and RNA synthesis block structural changes that accompany long-term heterosynaptic plasticity in Aplysia.. Neuron.

[12] Bailey CH, Kandel ER (1993). Structural changes accompanying memory storage.. Annu Rev Physiol.

[13] Malenka RC, Nicoll RA (1993). NMDA-receptor-dependent synaptic plasticity: multiple forms and mechanisms.. Trends Neurosci.

[14] Arancio O, Kandel ER, Hawkins RD (1995). Activity-dependent long-term enhancement of transmitter release by presynaptic 3′,5′-cyclic GMP in cultured hippocampal neurons.. Nature.

[15] Zakharenko SS, Zablow L, Siegelbaum SA (2001). Visualization of changes in presynaptic function during long-term synaptic plasticity.. Nat Neurosci.

[16] Toni N, Buchs PA, Nikonenko I, Povilaitite P, Parisi L (2001). Remodeling of synaptic membranes after induction of long-term potentiation.. J Neurosci.

[17] Nikonenko I, Jourdain P, Muller D (2003). Presynaptic remodeling contributes to activity-dependent synaptogenesis.. J Neurosci.

[18] Schuman EM, Madison DV (1991). A requirement for the intercellular messenger nitric oxide in long-term potentiation.. Science.

[19] Zhuo M, Hu Y, Schultz C, Kandel ER, Hawkins RD (1994). Role of guanylyl cyclase and cGMP-dependent protein kinase in long-term potentiation.. Nature.

[20] Arancio O, Kiebler M, Lee CJ, Lev-Ram V, Tsien RY (1996). Nitric oxide acts directly in the presynaptic neuron to produce long- term potentiation in cultured hippocampal neurons.. Cell.

[21] Son H, Lu YF, Zhuo M, Arancio O, Kandel ER (1998). The specific role of cGMP in hippocampal LTP.. Learn Mem.

[22] Lu YF, Kandel ER, Hawkins RD (1999). Nitric oxide signaling contributes to late-phase LTP and CREB phosphorylation in the hippocampus.. J Neurosci.

[23] Monfort P, Munoz MD, Kosenko E, Felipo V (2002). Long-term potentiation in hippocampus involves sequential activation of soluble guanylate cyclase, cGMP-dependent protein kinase, and cGMP- degrading phosphodiesterase.. J Neurosci.

[24] Antonova I, Arancio O, Trillat AC, Wang HG, Zablow L (2001). Rapid increase in clusters of presynaptic proteins at onset of long-lasting potentiation.. Science.

[25] Ninan I, Liu S, Rabinowitz D, Arancio O (2006). Early presynaptic changes during plasticity in cultured hippocampal neurons.. Embo J.

[26] Wang HG, Lu FM, Jin I, Udo H, Kandel ER (2005). Presynaptic and postsynaptic roles of NO, cGK, and RhoA in long-lasting potentiation and aggregation of synaptic proteins.. Neuron.

[27] Schafe GE, Bauer EP, Rosis S, Farb CR, Rodrigues SM (2005). Memory consolidation of Pavlovian fear conditioning requires nitric oxide signaling in the lateral amygdala.. Eur J Neurosci.

[28] Ota KT, Pierre VJ, Ploski JE, Queen K, Schafe GE (2008). The NO-cGMP-PKG signaling pathway regulates synaptic plasticity and fear memory consolidation in the lateral amygdala via activation of ERK/MAP kinase.. Learn Mem.

[29] Rumpel S, LeDoux J, Zador A, Malinow R (2005). Postsynaptic receptor trafficking underlying a form of associative learning.. Science.

[30] Yeh SH, Mao SC, Lin HC, Gean PW (2006). Synaptic expression of glutamate receptor after encoding of fear memory in the rat amygdala.. Mol Pharmacol.

[31] Nithianantharajah J, Murphy M (2008). Auditory specific fear conditioning results in increased levels of synaptophysin in the basolateral amygdala.. Neurobiol Learn Mem.

[32] Overeem KA, Ota KT, Monsey MS, Ploski JE, Schafe GE (2010). A Role for Nitric Oxide-Driven Retrograde Signaling in the Consolidation of a Fear Memory.. Front Behav Neurosci.

[33] Farb CR, Aoki C, Ledoux JE (1995). Differential localization of NMDA and AMPA receptor subunits in the lateral and basal nuclei of the amygdala: a light and electron microscopic study.. J Comp Neurol.

[34] Thiel G (1993). Synapsin I, synapsin II, and synaptophysin: marker proteins of synaptic vesicles.. Brain Pathol.

[35] Rodrigues SM, Schafe GE, LeDoux JE (2001). Intraamygdala blockade of the NR2B subunit of the NMDA receptor disrupts the acquisition but not the expression of fear conditioning.. J Neuroscience.

[36] Schafe GE, Atkins CM, Swank MW, Bauer EP, Sweatt JD (2000). Activation of ERK/MAP kinase in the amygdala is required for memory consolidation of pavlovian fear conditioning.. J Neurosci.

[37] Ninan I, Arancio O (2004). Presynaptic CaMKII is necessary for synaptic plasticity in cultured hippocampal neurons.. Neuron.

[38] Lamprecht R, Farb CR, LeDoux JE (2002). Fear memory formation involves p190 RhoGAP and ROCK proteins through a GRB2-mediated complex.. Neuron.

[39] Ressler KJ, Paschall G, Zhou XL, Davis M (2002). Regulation of synaptic plasticity genes during consolidation of fear conditioning.. J Neurosci.

[40] Lamprecht R, Farb CR, Rodrigues SM, LeDoux JE (2006). Fear conditioning drives profilin into amygdala dendritic spines.. Nat Neurosci.

[41] Radley JJ, Johnson LR, Janssen WG, Martino J, Lamprecht R (2006). Associative Pavlovian conditioning leads to an increase in spinophilin-immunoreactive dendritic spines in the lateral amygdala.. Eur J Neurosci.

[42] Gale GD, Anagnostaras SG, Godsil BP, Mitchell S, Nozawa T (2004). Role of the basolateral amygdala in the storage of fear memories across the adult lifetime of rats.. J Neurosci.

[43] Apergis-Schoute AM, Debiec J, Doyere V, LeDoux JE, Schafe GE (2005). Auditory fear conditioning and long-term potentiation in the lateral amygdala require ERK/MAP kinase signaling in the auditory thalamus: a role for presynaptic plasticity in the fear system.. J Neurosci.

[44] Ota KT, Monsey MS, Wu MS, Young GJ, Schafe GE (2010). Synaptic Plasticity and NO-cGMP-PKG Signaling Coordinately Regulate ERK-driven Gene Expression in the Lateral Amygdala and in the Auditory Thalamus following Pavlovian Fear Conditioning.. Learn Mem.

[45] Rodrigues SM, Farb CR, Bauer EP, LeDoux JE, Schafe GE (2004). Pavlovian fear conditioning regulates Thr286 autophosphorylation of Ca2+/calmodulin-dependent protein kinase II at lateral amygdala synapses.. J Neurosci.

[46] Zhuang S, Nguyen GT, Chen Y, Gudi T, Eigenthaler M (2004). Vasodilator-stimulated phosphoprotein activation of serum-response element-dependent transcription occurs downstream of RhoA and is inhibited by cGMP-dependent protein kinase phosphorylation.. J Biol Chem.

[47] Gudi T, Chen JC, Casteel DE, Seasholtz TM, Boss GR (2002). cGMP-dependent protein kinase inhibits serum-response element-dependent transcription by inhibiting rho activation and functions.. J Biol Chem.

[48] Etienne-Manneville S, Hall A (2002). Rho GTPases in cell biology.. Nature.

[49] Luo L (2002). Actin cytoskeleton regulation in neuronal morphogenesis and structural plasticity.. Annu Rev Cell Dev Biol.

[50] Smolenski A, Bachmann C, Reinhard K, Honig-Liedl P, Jarchau T (1998). Analysis and regulation of vasodilator-stimulated phosphoprotein serine 239 phosphorylation in vitro and in intact cells using a phosphospecific monoclonal antibody.. J Biol Chem.

[51] Fanselow MS (1980). Conditioned and unconditional components of post-shock freezing.. Pavlov J Biol Sci.

