# Supplementary figures and images for: Synaptic Plasticity and NO-cGMP-PKG Signaling Regulate Pre- and Postsynaptic Alterations at Rat Lateral Amygdala Synapses Following Fear Conditioning
Source: PLoS One. 2010 Jun 21;5(6):e11236. doi: 10.1371/journal.pone.0011236 (PMC2888610; doi:10.1371/journal.pone.0011236)

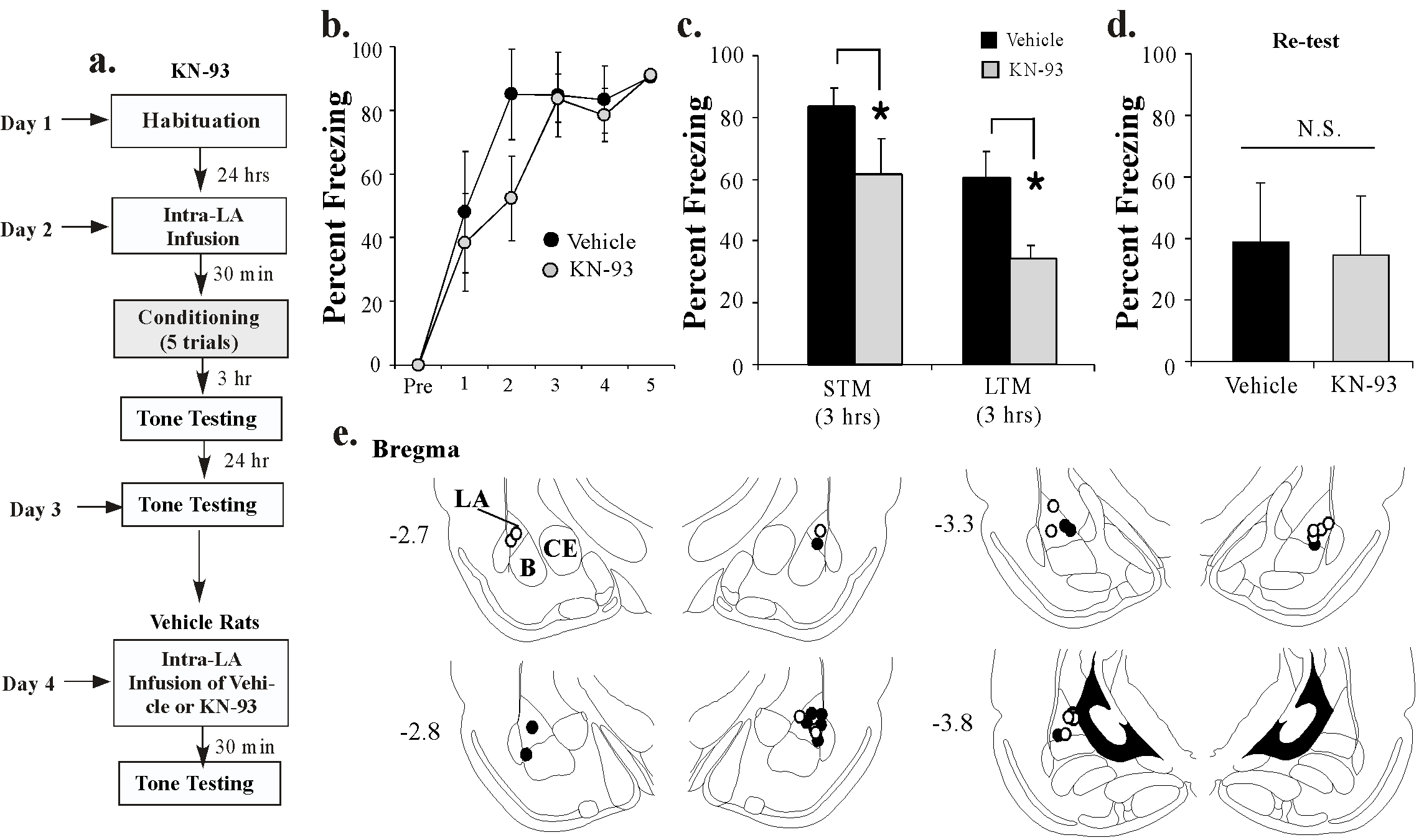

Supplement: Figure S1 — Inhibition of CaMKII in the LA impairs the acquisition, but not expression, of auditory fear memory. (A) Schematic of the behavioral protocol. Rats were given intra-LA infusion of either the vehicle or KN-93 (1 ug). Thirty minutes later they were trained with five tone-shock pairings, then tested for retention of auditory fear conditioning at 3 and 24 hrs following conditioning. Twenty-four hours after the LTM test, rats that had originally been infused with vehicle were re-infused with either ACSF (n = 3) or 1 ug KN-93 (n = 4), then re-tested for auditory fear memory 30 minutes later. (B)Mean (+/− SEM) post-shock freezing between conditioning trials in rats given intra-LA infusions of ACSF (vehicle; n = 7) or 1 ug KN-93 (n = 8). (C) Mean (±SEM) auditory fear memory assessed at 3 hr (STM) and 24 hrs (LTM) following conditioning. (D) Mean (±SEM) auditory fear memory assessed at 30 minutes following re-infusion. Histological verification of cannula placements for rats infused with 1 ug KN-93 (white circles) or ACSF vehicle (black circles). Panels adapted from Paxinos and Watson (1997). (*) p<0.05 relative to vehicle. (9.24 MB TIF) [file pone.0011236.s001.tif]
