# Supplementary material for: Synaptic Plasticity and NO-cGMP-PKG Signaling Regulate Pre- and Postsynaptic Alterations at Rat Lateral Amygdala Synapses Following Fear Conditioning
Source: PLoS One. 2010 Jun 21;5(6):e11236. doi: 10.1371/journal.pone.0011236 (PMC2888610; doi:10.1371/journal.pone.0011236)
Supplement: Methods & Analysis S1 — Supplemental methods and analysis for Figure S1. (0.03 MB DOC) [file pone.0011236.s002.doc]

**Supplemental Methods & Analysis (S1)**

A previous study has shown that intra-LA infusion of the CaMKII inhibitor KN-62 impairs fear memory acquisition without impairing routine synaptic transmission in the LA; that is, pre-training intra-LA infusions of KN-62 impair both STM and LTM of auditory fear conditioning, while pre-testing infusions have no effect on retrieval of fear memory [45]. In the present study, we have used the CaMKII inhibitor KN-93,which, to our knowledge, has not previously been examined in auditory fear conditioning. Accordingly, we ran a separate behavioral experiment to verify that intra-LA infusion of KN-93 impairs fear memory acquisition without having effects on routine synaptic transmission in the LA.

Rats were cannulated as described in the main Methods section. For two days prior to conditioning, rats were habituated to the conditioning chamber and to dummy cannula removal for a minimum of 10 minutes. The following day, rats were given intra-LA infusion of either ACSF (0.5 μL; 0.25 μL/min) or KN-93 (1 μg/side in 0.5 μL; 0.25 μL/min). The ACSF and KN-93 were prepared as described in the main Methods section. Injectors remained in the cannulas for 1 minute after drug infusion to allow diffusion of the drug from the tip. Thirty minutes following drug infusion, rats were trained with 5 conditioning trials consisting of a 20 sec, 5kHz, 75dB tone that co-terminated with a 1.0 sec, 0.5 mA foot shock, respectively (ITI=120 sec). Testing for conditioned fear to the tone occurred at 3 and 24 hours following training. For each test, rats were placed in a distinctive environment that was dark and consisted of a flat black plastic floor that had been washed with a peppermint-scented soap. For the 3 hour test, rats were exposed to 5 conditioned stimulus (CS) tones (5kHz, 75dB, 20 sec). For the 24 hour test, rats were exposed to 10 CS tones. For each tone test, we measured the rats’ freezing behavior, defined as a lack of all movement with the exception of that required for respiration, and expressed this measure as a percentage of the total CS presentation time. Freezing was calculated from activity counts measured automatically during each CS presentation by Coulbourne Instruments Activity Monitors (Model # H10-24A) mounted at the top of each of the behavioral chambers. For each memory test, freezing scores were averaged across trials for each rat. All data were analyzed with ANOVAS or t-tests. Differences were considered significant if P<0.05. At the end of the behavioral experiment, rats were sacrificed by an overdose of chloral hydrate (600mg/kg) and perfused with 0.9% saline followed by 10% buffered formalin. Nissl staining and light microscopy were used to verify the location of the cannula tips within the amygdala.

The findings of the behavioral experiment can be seen in Figure S1. Rats infused with KN-93 exhibited intact post-shock freezing during training that did not significantly differ from vehicle infused controls (Fig. S1B). The ANOVA for post-shock freezing scores showed only a significant effect of trial [F(4,65)=5.09, p<0.001]; the effect for drug [F(1,65)=1.62] and the drug by trial interaction [F(4,65)=0.62] were not significant. In contrast, KN-93-infused rats tested for auditory fear memory 3 hours following conditioning were found to have impaired short-term memory relative to vehicle-infused rats [t(13)=2.24, p<0.05] (Fig. S1C). This difference remained at 24 hours after training, where those animals given intra-LA infusions of KN-93 again showed significantly decreased levels of freezing relative to those that received vehicle infusions [t(13)=3.17, p<0.01] (Fig. S1C).

Our initial experiment showed that pre-training infusion of KN-93 impairs auditory fear memory formation. The observation of intact post-shock freezing (Fig. S1B) suggests that KN-93 does not nonspecifically interfere with shock processing during the training session. However, it remains possible that the memory impairment observed at 3 and 24 hours (Fig. S1C) could reflect impairments in auditory processing in the LA by the KN-93 infusion. To address this concern, the vehicle-infused rats from the initial behavioral experiment were divided into comparable groups based on their 24 hour memory score and re-infused with either vehicle or KN-93 (1 μg) one day after the LTM test. They were then retested for fear memory 30 minutes after infusion (Fig. S1D). Thus, these initially vehicle-infused animals that were conditioned drug-free were tested for auditory fear memory retention either drug-free or under the influence of the KN-93. The findings revealed that both groups displayed equivalent levels of freezing [t(5)=0.14]. Thus, it is unlikely that the memory impairment observed during the original 3 and 24 hour tests can be attributable to impairments in fear expression in the KN-93 group, suggesting that inhibition of CaMKII in the LA impairs the acquisition, but not the expression, of auditory fear memory.
